# Thermodynamic Concepts in the Study of Microbial Populations: Age Structure in *Plasmodium falciparum* Infected Red Blood Cells

**DOI:** 10.1371/journal.pone.0026690

**Published:** 2011-10-31

**Authors:** Jordi Ferrer, Clara Prats, Daniel López, Jaume Vidal-Mas, Domingo Gargallo-Viola, Antonio Guglietta, Antoni Giró

**Affiliations:** 1 Departament de Física i Enginyeria Nuclear, Escola Superior d'Agricultura de Barcelona, Universitat Politècnica de Catalunya, Castelldefels, Spain; 2 Tres Cantos Medicines Development Campus, GlaxoSmithKline, Tres Cantos, Spain; 3 Pharmacology and Safety Department, Ferrer Grupo Research Center, Barcelona, Spain; Agency for Science, Technology and Research - Singapore Immunology Network, Singapore

## Abstract

Variability is a hallmark of microbial systems. On the one hand, microbes are subject to environmental heterogeneity and undergo changeable conditions in their immediate surroundings. On the other hand, microbial populations exhibit high cellular diversity. The relation between microbial diversity and variability of population dynamics is difficult to assess. This connection can be quantitatively studied from a perspective that combines *in silico* models and thermodynamic methods and interpretations. The infection process of *Plasmodium falciparum* parasitizing human red blood cells under laboratory cultivation conditions is used to illustrate the potential of Individual-based models in the context of predictive microbiology and parasitology. Experimental data from several *in vitro* cultures are compared to the outcome of an individual-based model and analysed from a thermodynamic perspective. This approach allows distinguishing between intrinsic and external constraints that give rise to the diversity in the infection forms, and it provides a criterion to quantitatively define transient and stationary regimes in the culture. Increasing the ability of models to discriminate between different states of microbial populations enhances their predictive capability which finally leads to a better the control over culture systems. The strategy here presented is of general application and it can substantially improve modelling of other types of microbial communities.

## Introduction

Malaria is an infectious disease caused by parasites of the genus *Plasmodium* propagating within Red Blood Cells (RBCs). Human malaria infections exhibit high genetic and phenotypical diversity [1]. Monoclonal *in vitro* cultures of *Plasmodium falciparum* (*Pf*) also comprise highly heterogeneous populations whose diversity increases with time in culture [2], [3]. These cultures exhibit a significant variability in their performance, which hinders the evaluation of their response to drug treatments [4]. The main purpose of this contribution is to provide tools to measure the diversity of *Pf in vitro* cultures and to estimate their variance.

Recent advances in single-cell technology have enabled microbiologists to be increasingly aware of microbial heterogeneity [5], [6], [7]. One of the current challenges in microbiology is to tackle the connection between individual traits of cells and the collective behaviour of populations, and to channel this insight into a better control of microbial systems [8].

Computational modelling is required to interpret the huge amount of newly available biological data and to integrate molecular, cellular and population levels of description [9], [10]. *In silico* modelling is also used in epidemiology to better control and to predict the spreading of infectious agents [11]. Individual-based Models (IbMs) are computational models that describe how the behaviour and properties of collectives emerge from the rules governing individuals and their local interactions [12]. IbMs in microbiology usually focus on how cellular characteristics and interactions mediated through molecular diffusion shape the structure and dynamics of microbial communities [13], [14], [15], [16].

Thermodynamics in its broadest sense is a body of methods and interpretations that describe how macroscopic systems change when they interact with one another or with their surroundings. Biological thermodynamics quantitatively studies energy transformations in biological processes [17]. In the context of theoretical ecology, non-equilibrium thermodynamics is widely used for explaining organization of communities [18], [19], [20], [21]. In computational microbiology, thermodynamics is usually employed to study physico-chemical interactions at a molecular level [22], metabolic or gene-regulatory networks at a whole-cell level [23] and population dynamics at a system level [24].

This contribution combines the use of IbMs with a thermodynamic perspective to analyse the relation between diversity of a population and variability of its collective behaviour. Specifically, we study the population structure and infection dynamics of experimental *Pf in vitro* cultures under standard conditions [25], we use the IbM INDISIM-RBC [26], [27] to describe them, and we borrow some thermodynamic concepts to quantitatively analyse them. We focus on two easily measurable magnitudes (age of RBCs and infection state of IRBCs) and explore whether it can be expected that two cultures submitted to the same cultivation protocol but showing different population structures will present similar infection dynamics, and if so, to what extent. As a result, we propose a quantitative criterion for discriminating between transient and stationary states in these cultures. This distinction is important in the context of drug treatment: when testing the effect of any external agent over a culture, it must be granted that the tested and control cultures are in the same state.

However, this paper does not attempt to quantitatively account for the entire biological thermodynamics of malaria cultivation: the formal treatment of the thermodynamic forces driving cellular metabolism, parasite invasion and cellular growth is beyond the scope of this study. Nor do we claim to capture all the complexity of real cultures: for instance, we do not tackle gametocyte formation, which is an important indicator of the healthiness of cultures [28], [29]. Withal, the model quantitatively predicts the behaviour of *in vitro* cultures, it allows a better assessment of infection dynamics and it has favoured the enhancement of culturing protocols, including semi-automated cultivation in suspension.

## Methods

### *In vitro* cultures of *Plasmodium falciparum-*infected red blood cells

#### This section presents the experimental systems under study and their measured features

Continuous cultivation enables the long-term propagation of the parasite in repeated life cycles where merozoites (infective form of *Pf*) invade RBCs and reproduce asexually. Current standard cultivation protocols are static cultures with periodic renewals of culture medium and sub-cultivations (replacement of a fraction of the RBC population with fresh healthy cells) that ensure the availability of nutrients and of targets susceptible to infection [30]. An alternative to static cultures is maintaining RBCs in suspension through the continual agitation of the system [31], [32].

This contribution employs data extracted from several experimental trials carried out during the period 2005-2009 by the Experimental Microbiology Group from GlaxoSmithKline [26], [27]. IRBCs were cultured following the standard protocols proposed by the Malaria Research and Reference Reagent Resource Centre [25], with slight modifications (*i.e.* blood source, parasite strain, protocol for sub-cultivation and medium renewal).

Human RBCs for malaria cultivation were obtained from blood not usable for blood transfusions, kept frozen at -80°C and subsequently stored at temperatures that ranged from 1°C to 4°C. RBC storage (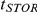) affects their suitability: susceptibility to infection decreases with RBC age (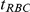) as merozoites preferentially invade young fitter cells [33], [34]. Cultures were maintained over periods that range from 10 to 30 days, although it has been reported that cultures can be maintained over several months [32].

Samples of the IRBC population were extracted periodically and examined through optical microscopy. We distinguished four infection forms (*f_INF_*) along the 48-hour reproductive cycle of the parasite and their characteristic durations: ring stage (*t_R_* ∼ 18 hours), throphozoite stage (*t_T_* ∼ 17 hours), early schizont stage (*t_S_* ∼ 7 hours) and late schizont stage (*t_F_* ∼ 6 hours). This discriminating criterion is useful to study the fragility of mature forms. We also recognized multiply-infected IRBCs.

We analyzed the dynamics of the distribution of infection forms *q*(*f_INF_*). In theory, the time-after-invasion of IRBCs (*t_INF_*) can be related to their hemoglobin content or to the presence and abundance of certain membrane proteins [35], however, the distribution of post-invasion times *q*(*t_INF_*) could not be certainly measured.

A detailed description of the experimental trials can be found in the Supporting Information file Text S1.

### Thermodynamic concepts

This section provides a brief introduction to thermodynamics notions and describes how we adapt them to the context of population dynamics, even at the risk of abusing of terminology.

Systems that comprise a large number of elements (such as microbial populations) can be described at two levels: the *microscale* (that of individuals) and the *macroscale* (that of populations). The macroscopic state of any such system can be defined by measuring a set of whole-system variables, while assessing its microscopic configuration would entail measuring every characteristic of every individual at one time, which is unfeasible in practice. The correspondence between macroscopic states and microscopic configurations is not one to one, as many arrangements at the microscale are compatible with a single macroscopic state. For instance, a completely synchronous malaria culture requires that all the IRBCs are in the same infection stage, while an asynchronous culture is compatible with many configurations of infection stages of RBCs. The thermodynamic property *entropy* can be used to measure the number of microstates compatible with an observed macrostate.

Thermodynamics distinguish between *equilibrium states*, *stationary states* and *variable states* (e.*g. transient states*), and between *time-reversible* and *irreversible processes. S*trictly speaking, all biological systems are open (*i.e.* they exchange matter with their surroundings) dissipative (*i.e.* they release heat to their surroundings) systems far from the thermodynamic equilibrium, in variable states and undergoing irreversible processes [18]. Withal, apparently unchanging stages may occur when a system is maintained under constant constraints (*e.g.* homeostasis at a cellular level, or balanced growth in a chemostat at a population level). We say that such a system is in a stationary regime or (weak) stationary state. In sum, the definition of the state of a system makes sense only once the time scale and resolution of our observations have been specified.

There are four fundamental laws of thermodynamics. The zero law establishes an equivalence relation between macroscopic systems and the first law is the conservation of energy. Both laws are relatively straightforward. The second law specifies the “arrow of time” in which macroscopic processes occur and it deserves further attention. The third law concerns the behaviour of systems near the absolute zero of temperature and it does not concern the present discussion.

The second law of thermodynamics states that any macroscopic system tends towards a state of equilibrium (or towards a stationary state compatible with external constraints) after a transient, while its entropy (*S*) tends to increase. The concept of entropy can be generalized to measure the diversity (degree of heterogeneity) of any categorical data set [36], for instance, the distribution of a variable feature among individual cells.

In the context of population ecology, the prevalence of stationary states can be interpreted as the tendency of communities towards their maximum diversity [19], [37]. At any time, the diversity of the population can be measured with the Shannon index *S_x_*, which represents the diversity of a population regarding the individual trait (*x*):
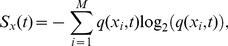
(1)where the sub-index *i* indicates each of the predefined categories by which *x* may be sorted, *M* is the number of categories defined this way, and *q(x_i_,t)* is the distribution function or frequency of occurrence of each category within the population. This index takes into account the number of categories as well as their evenness. Therefore, the index is increased either by considering additional categories, or by increasing the evenness of *q(x_i_,t).* Equation 1 expresses diversity in units of information: *bits* [36], [38].

Diversity of a population is the trade-off between the intrinsic variability of individuals and the homogenizing effect of interactions and external constraints. If no constraints are imposed on the system beyond the normalization of 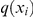 (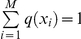), the uniform distribution is the one that leads to maximum diversity 
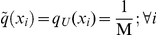
. Otherwise, variational principles can be used to define 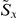, the maximum value of 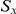 that is compatible with a set of observed constraints. Setting 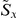 is analogous to determining 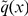, the expected distribution of *x* among the population in the stationary state.

An extended interpretation of the second law of thermodynamics, expressed as the maximum diversity formalism described above, provides a strong insight into population diversity [39]. As long as the external constraints on it are kept constant, a population may eventually reach and remain in a “stationary” state, characterized by 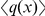, the distribution 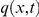 averaged over time. Whenever this occurs, the observed instantaneous diversity averaged over time through the stationary state, 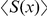, ideally takes the same value as 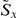, the diversity computed through the maximum diversity formalism [38].

Following the discussion above, we propose a practical method for assessing whether a system may have reached a (weak) stationary state: to check if its instant diversity 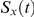 significantly differs from the prevalent diversity 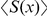, in particular, we assume that the system may have reached stationarity only if 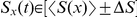, where 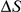 is the standard deviation of the diversity in the stationary state.

### Individual-based Modelling

INDISIM is an IbM methodology designed to address microbial communities under specific controlled environmental conditions [40]. INDISIM models the evolution of a microbial population at discrete time steps with deterministic and stochastic rules applied to each cell to its local environment, and to the system as a whole. It defines and controls a matrix representing a population of cells in an explicit discrete space that captures the local characteristics of the immediate surroundings of each microbial cell. A set of boundary conditions and holistic rules represent the external constraints on the system.

INDISIM-RBC was developed to study the *in vitro* cultivation of *Pf* IRBCs [26], [27]. It accounts for ∼10^6^ microbial cells set in a spatially explicit representation of a small fraction of the culture system. It does not provide an exhaustive description of the cellular metabolism of RBCs or the infection cycle of IRBCs, but rather a schematic summary of their relevant features: RBC life cycle, IRBC infection cycle and merozoite spreading and decay. An outlined description of the model can be found in the Supporting Information file Text S2.

The bulk of the simulations have been carried out to study the effect that different external factors (storage period of RBCs, diversity of the inoculated IRBCs and sub-cultivation protocol) have on the infection dynamics and they have been compared to the experimental work presented above. The parameters used to fit the model and experiments were macroscopic quantities such as the parasitaemia or fraction of infected cells 
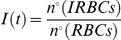
, the ratio of each infection stage, the fraction and degree of multiple infections, and the growth ratio per infection cycle (*GR*).

INDISIM-RBC is used here to track the population structure of cultures: the age distribution of RBCs 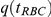, and the distribution of infection stages 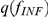 and post-invasion times 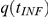 of IRBCs.

At any time *t*, the diversity of the modelled IRBC population with regards to the degree of synchronism of the infection can be defined in terms of 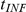 and measured in bits:
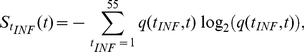
(2)where IRBCs are sorted into 55 one-hour bins to account for a potential increased duration of the 48-hour infection cycle due to individual variability. When only the normalization of 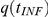 is imposed (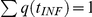), the maximum diversity is found when IRBCs are uniformly distributed: 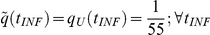. The resulting diversity takes the value 
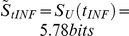
.

Alternatively, the diversity defined in terms of the four observable infection stages *f_INF_* can be used. This magnitude serves to compare simulation results with experimental data:
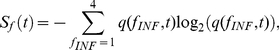
(3)where IRBCs are sorted into the 4 stages that were singled out experimentally. The maximum diversity attainable with four infection stages is achieved when all of them are equiprobable: 
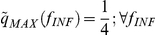
 and 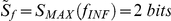.

However, the diversity obtained when imposing maximum evenness to the population of IRBCs (for a uniform distribution of 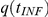) is below this value because each infection stage has a characteristic duration. Consequently, infection stages do not appear with the same frequency but with one weighted by their relative durations. The expected diversity that corresponds to maximum evenness of IRBCs occurs when: 
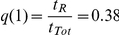
; 
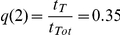
; 
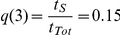
 and 
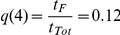
 (with *f* = 1 ring stage; *f* = 2 trophozoite; *f* = 3 early schizont stage; *f* = 4 late schizont stage), and it takes the value 
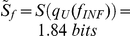
.

## Results

Experimental cultures of IRBCs have been set from different initial conditions and have been successfully maintained for up to thirty days. They perform with a wide range of infection yields depending on the initial conditions and on the culturing protocol. All cultures exhibit increasingly asynchronous populations of IRBCs mainly comprised by ring forms. INDISIM-RBC simulations have been compared to these experiments.

**Figure 1:**
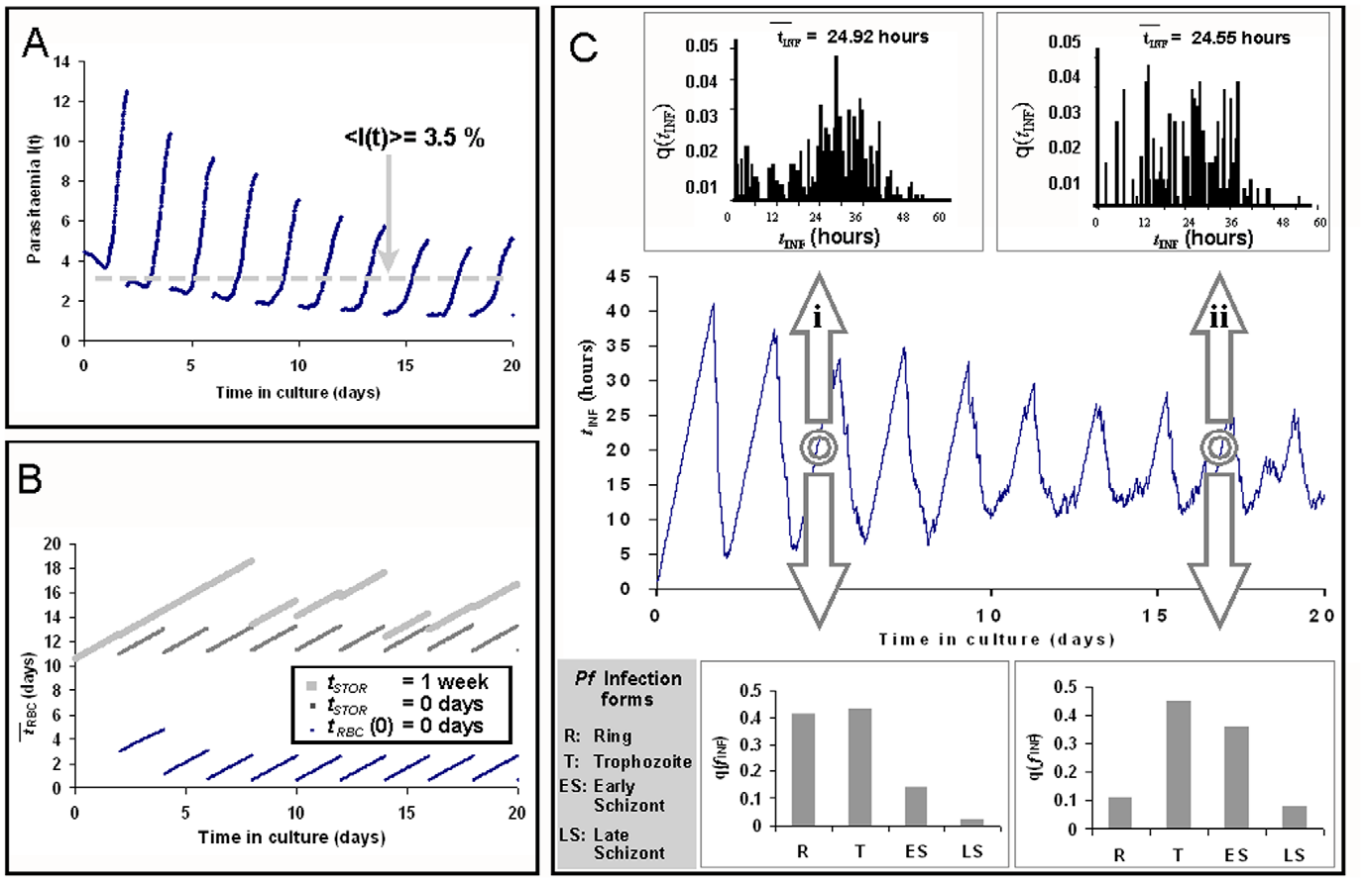
Outcome of simulated cultures. Evolution of a standard static culture with synchronous inoculum (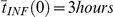), sub-cultivations every two days and one week storage of the blood source. A) Percentage of infected forms. B) Evolution of the mean age of RBCs in culture (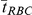) through time in culture (days) for different storage periods of the blood source (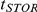). The label 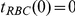 indicates an inoculum comprised by RBCs with age 0. C) Mean post-invasion time of IRBCs in culture (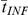). Samples of the culture with similar 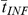 show different distributions (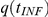 and 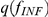) at day 4 (i) and at day 12 (ii), revealing that the population becomes increasingly desynchronised.

Figure 1 shows the simulated evolution of a culture with daily medium renewal and under a 2-day sub-cultivation protocol. Figure 1A shows that the performance of the culture gradually decreases until reaching an average parasitaemia 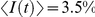 after 10 days in culture. Figure 1B compares the mean age of RBCs for different storage periods prior to sub-cultivation. Figure 1C shows that the population of IRBCs gradually desynchronises until reaching a certain diversity, which is maintained from then on. This overall behaviour is modulated by periodic oscillations that follow the 48-hour infection cycle.

### Performance of the simulated cultures

INDISIM-RBC is used to account for global and local restrictions on 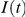 imposed by the availability of substrate and of fresh RBCs. We can distinguish two factors contributing to the shortage of RBCs. The first factor is local availability of RBCs: parasites must have a minimum number of potential hosts at range after every infection cycle because they can not survive in the extracellular medium for long. This limitation to the spreading of the parasite, together with the restrictions caused by substrate diffusion in static cultures, is discussed in Ferrer *et al.* [27].

The second factor is quality of RBCs: the storage of blood prior to cultivation affects the culture performance because it makes RBCs less susceptible to infection as merozoites invade more easily younger and fitter RBCs. Figure 1B presents how the mean age of RBCs in culture (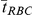) is affected by different storage periods (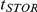). It shows i) that under standard culturing conditions and when sub-cultivations are performed every two days the RBC population is completely replaced by stored RBCs after five days approximately, and ii) that 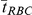 increases with 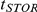.

**Figure 2:**
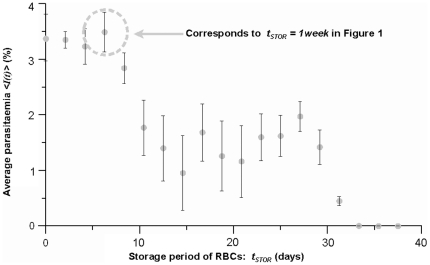
Simulated effect of the storage of RBCs on the culture performance. Average parasitaemia through the culture decreases with the storage time of RBCs. Grey dots show the mean values obtained from two sets of simulation trials that included sub-cultivations every 48 h, 72 h and 96 h. Errors bars reflect the variability in the simulations.

Figure 2 shows the effect of 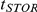 on the performance of the cultures. It reveals that storing the blood for up to a week has no practical effect on the culture performance, but after ten days of storage, the average parasitaemia is reduced to half of its maximum value. Storing the blood for more than a month makes cultivation unfeasible. These results are in accordance with experimental observations [33], [34]. They have been used to evaluate and predict the effect of different storage periods and sub-cultivation protocols on the performance of real cultures.

### Optimization of cultivation protocols

For low parasite loads and short time spans, unrestricted proliferation of the parasite can be assumed. The evolution of the parasitaemia can be fit to an exponential growth:
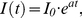
(4)where 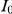 is the parasitaemia set after every sub-cultivation and 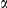 is the growth rate.

INDISIM-RBC outcomes were fitted to experimental data of the parasitaemia observed in daily samples of static (A) and suspended (B) cultures and used to predict the evolution of automated cultures operating with a fixed fraction of sub-cultivation (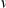). The characteristic values obtained this way are the parameters in Equation 4, 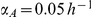 and 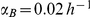. According to the model in Equation 4, the fraction of culture to be extracted at every sub-cultivation (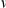) was determined through imposing that 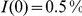 after each sub-harvesting, 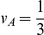 and 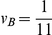 (see Supporting Information file Text S1).

**Figure 3:**
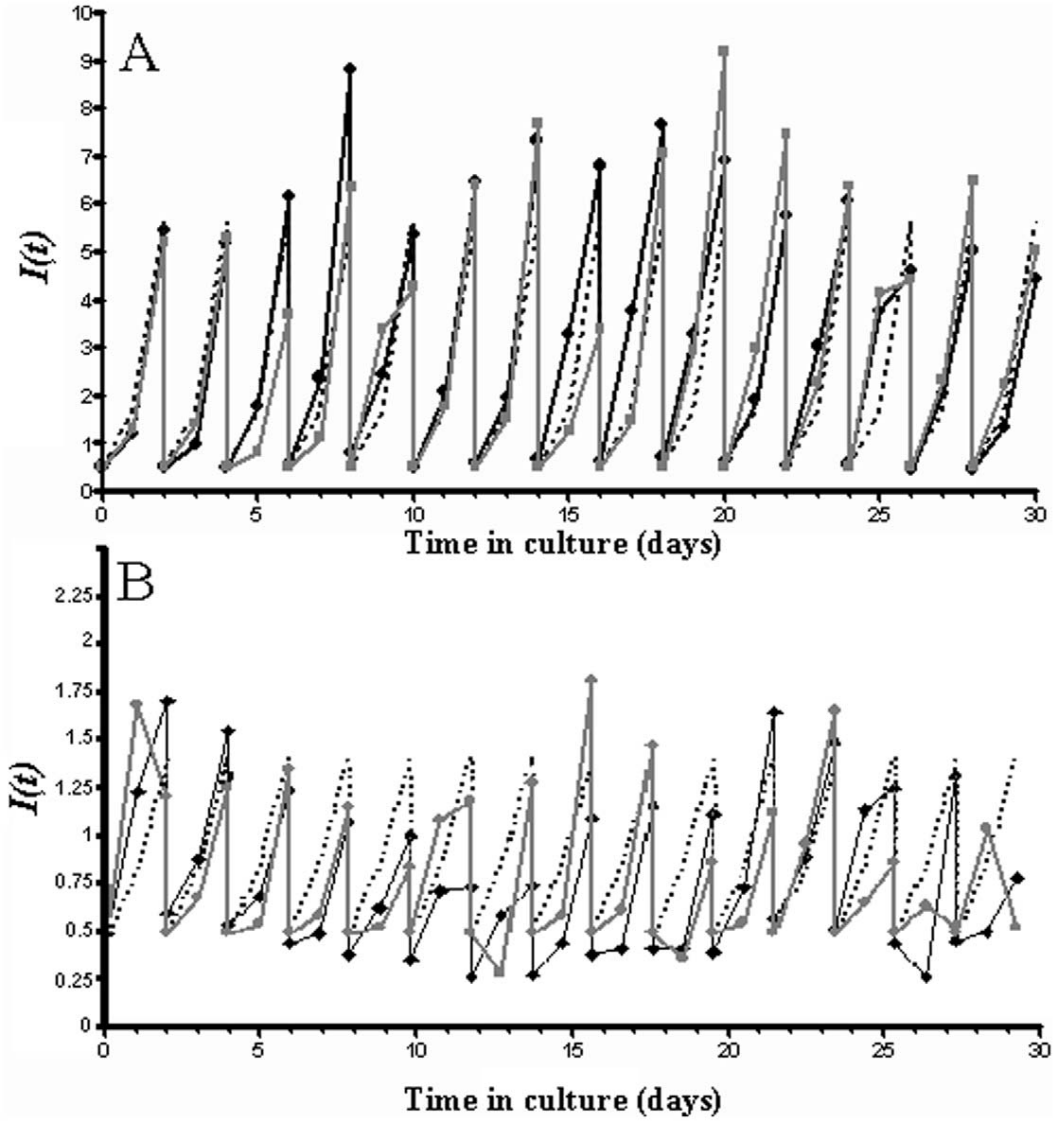
Course of the parasitaemia *I(t).* Daily samples in suspended (A) and static (B) long-term cultivation of *P. falciparum-*infected RBCs under standard conditions compared to the model predictions. Squares and grey solid line correspond to a culture with standard sub-cultivation protocol. Dotted line shows the simulated behaviour of automatized sub-cultivation with ratios. Black solid line shows the performance of real automatized cultures.

Figure 3 shows experimental performance of static and suspended cultures, compared to the INDISIM-RBC predictions and to the outcome of the proposed automated sub-cultivation protocol. An extended description of this experiment and of the methods used to fit simulations to experimental results can be found in the Supporting file Text S1.

### Population structure of the simulated cultures

INDISIM-RBC has been used to analyse the role of diversity of *P. falciparum* in *in vitro* cultures using the thermodynamic notion that tendency towards maximum diversity leads to stationary states under fixed constraints.

#### Evolution of the distribution of post-invasion time

The quantitative analysis of 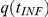 is put forward to present the methodology used to study the tendency of populations towards maximum diversity with INDISIM-RBC simulations, although these results could not be directly compared to experimental data. At any time *t*, the diversity of the IRBC population has been computed in terms of 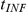 as stated in the Section *Methods*. The diversity computed through each simulation has been analyzed to obtain their average behaviour in the long-term (after more than a week culture), 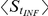. In addition, the maximum diversity attainable by the system (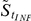) has been calculated by maximizing the diversity defined in Equation 2 under the external constraints imposed by the experiments. These values have been compared to study the dynamical state of the population.

**Figure 4:**
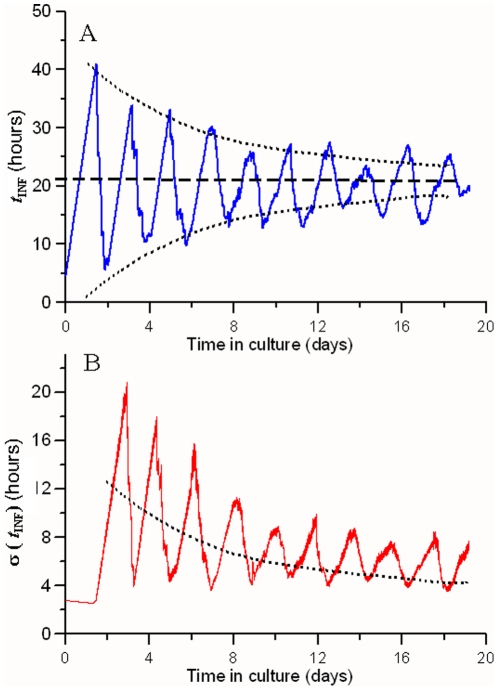
Simulated evolution of of post-invasion times. (A) mean value (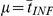) and (B) standard deviation (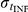) of the distribution of post-invasion times among a simulated population of IRBCs. Dashed line indicates the asymptotic trend towards: 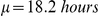. Dotted line depicts the correspondence between the envelope of *μ*and the mean value of *σ* ~ 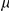 and *σ* are approximately 180° out of phase because *σ*reaches its maximum value when the generalized outburst of the parasite takes place; at that moment, 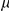 has a medium value because most of the IRBCs are at the end of their infection cycle while the next generation of parasites is already rising.

Figure 4 shows (A) the evolution of the mean post-invasion time 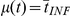 and (B) of the instantaneous standard deviation 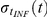 (dispersion of 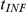 observed through the IRBC population) of a simulated static *in vitro* culture comprising young rings (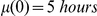, 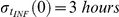), with a 1-week storage period and 2-day periodic sub-cultivations. A trend towards a certain average value of post-invasion times is observed in the long-term: 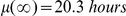. The standard deviation of 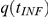 increases after the first infection outburst and it gradually decreases until reaching a minimum value, 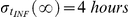. If we average the stationary values shown obtained from 10 simulations, we find that the mean value obtained for the mean post-invasion time and its temporal standard deviation (dispersion around the mean observed value) are: 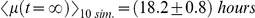 and 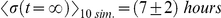, respectively, where the values after the 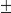 symbol indicate the standard deviation found when analyzing several simulations, 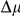 and 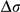, respectively (see Supporting Information file Chart S1).

**Figure 5:**
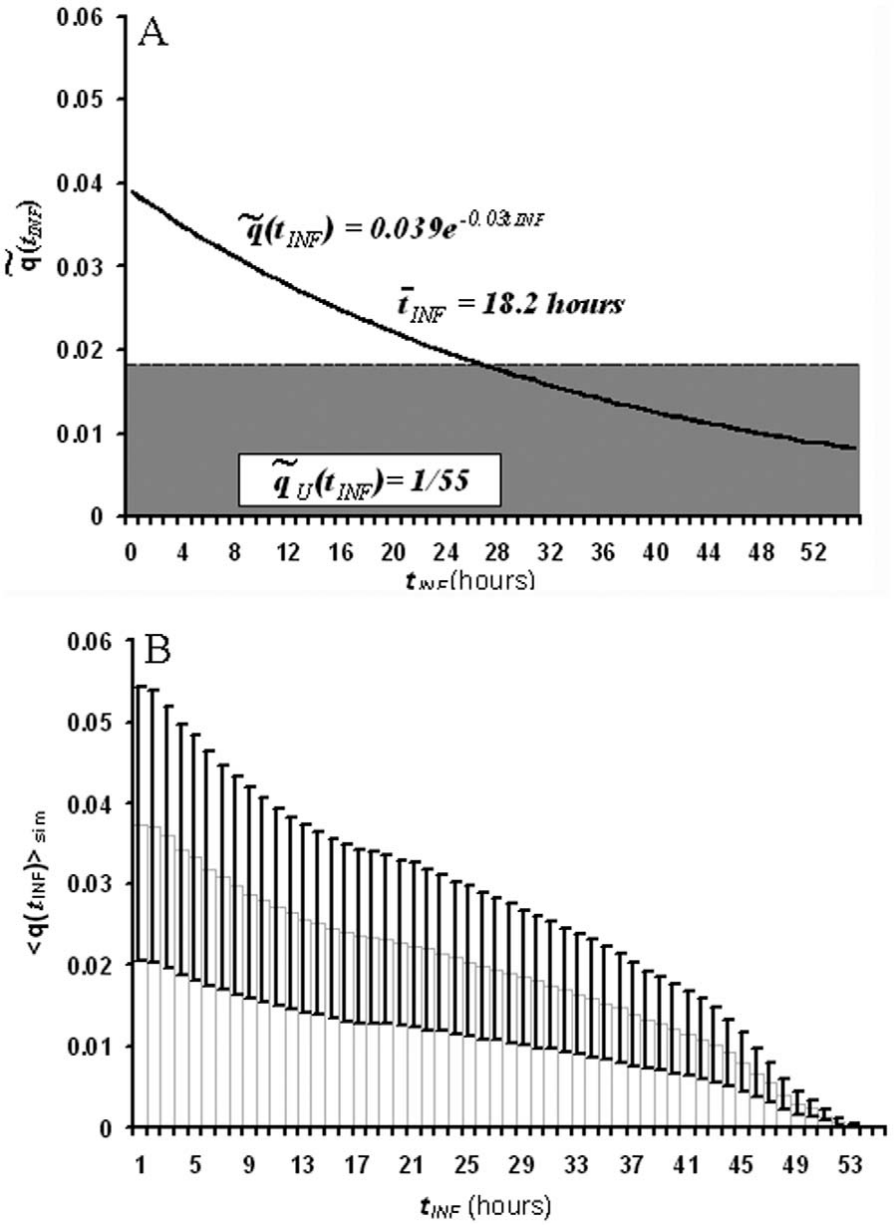
Expected and simulated distributions of post-invasion times 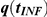. A) Expected theoretical distributions obtained from the optimization of system's diversity. Grey solid distribution: assuming a uniform distribution of IRBCs. Black line: imposing a fixed observed post-invasion time 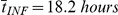. B) Simulated average distribution of post-invasion times 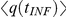 among the population of IRBCs in a static culture set from a rather synchronous inoculum (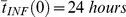) with sub-cultivation carried out every two days. Error bars indicate the standard deviation 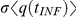 observed through 15 days in culture under the stationary regime.

Figure 5 compares the distributions 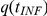
obtained by maximizing the diversity of the population and fixing the external constraints (Figure 5A), and by averaging over the simulation outcomes (Figure 5B). When the diversity 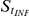 is maximized imposing only the normalization of 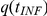, 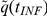 shows a uniform distribution 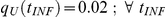. When the additional constraint of fixing the observed mean value of the post-invasion time averaged over the population is imposed, maximizing 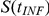 leads to an exponentially decaying function 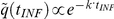. In particular, if we impose 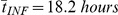, then 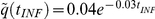 and its associated diversity is 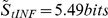.

In contrast, the distribution of post-invasion times averaged over 10 INDISIM-RBC simulations reproducing standard conditions shows a prevalent diversity 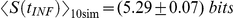, where the value after the symbol 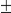 represents the prevalent standard deviation of the diversity (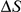), the dispersion observed in the simulation outcomes of the ten repetitions (see Supporting Information file Chart S1).

The diversity index observed in the simulations 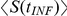 is smaller than 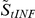 the diversity obtained by the maximization formalism when 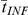
is imposed. This fact only indicates that our model does bind the system, as it imposes additional constraints on the population of IRBCs beyond fixing 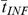 [39]. The comparison between the model predictions and what is found in reality is put forward in the next sections.

### Evolution of the distribution of infection stages and comparison with experimental results

The distribution of infection stages *q*(*f_INF_*) is more easily measured in experiments so it will be employed to analyse these constraints from now on instead of *q*(*t_INF_*).

**Figure 6:**
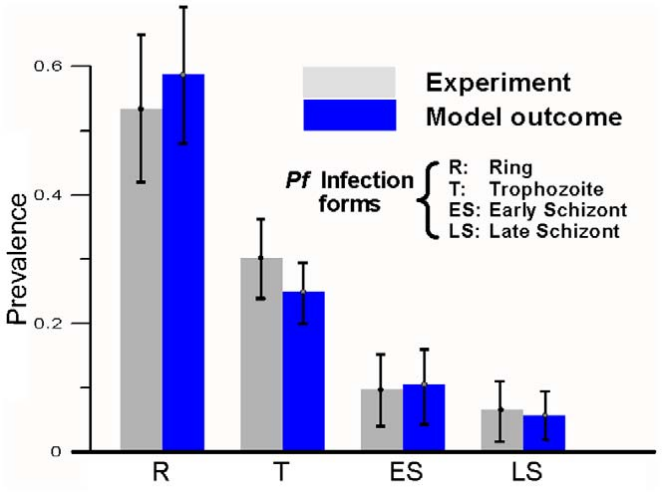
Experimental and simulated prevalence of infection forms 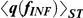. R  =  ring, T  =  trophozoite, ES  =  early schizont and LS  =  late schizont. Bars show the prevalent distributions averaged over twenty days for ten culture systems under the standard protocols, with sub-cultivations performed every two, three or four days and with different diversity of the inocula. Error bars correspond to the standard deviation observed through the different trials 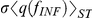.

Real cultures usually exhibit 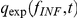 that vary with periodic oscillations in *t*. However, they also show a tendency towards a prevalent distribution 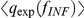 (see Figure 6). Even when cultures are set from asynchronous inocula (*i.e.* when 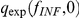 is uniform), 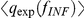 exhibits a preponderance of ring stages. This means that the population decreases its diversity as time goes by, which implies that additional factors are limiting the infection dynamics and shaping the distribution of infection forms.

At any time *t*, diversity is defined in terms of the four observable infection stages as stated in Equation 3. INDISIM-RBC reproduces this behaviour (see Figure 6) and it can be used to study the factors that shape 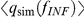 just as we did with post-invasion time. The prevalent distribution of infection forms observed in experimental cultures fits well the exponential function: 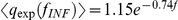. Its associated diversity takes the value 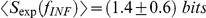. The attainment and analysis of these results are detailed in the following section. The factors that shape 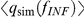 are presented next.

### Quantitative definition of transient and stationary states

Experimental cultures have been sampled daily to monitor the evolution of 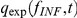. The instantaneous diversity 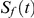 has been computed from each sample using Equation 3, and the prevalent distribution 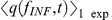 and diversity 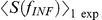 have been obtained by averaging 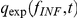 and 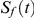 over time during each culture trial.

After considering various static culture trials with different initial and sub-cultivation conditions, it has been noted that 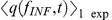 slightly varies from trial to trial (see additional information in the supporting file Text S1). Therefore, the diversity of infection forms under standard conditions is set to the prevalent diversity 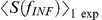 averaged over 29 experimental trials: 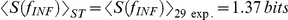. This value, together with the standard deviation of the distribution of infection forms 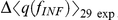, has been used to to define the threshold 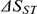:
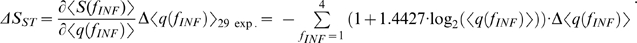
(5)


Assuming the values of 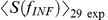 and 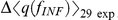 (shown in Figure 6 as error bars), we find: 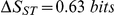.

The comparison between 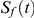 and 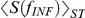 offers a quantitative criterion for discriminating the state of culture at any time *t*. In other words, 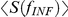 is an indicator of the dynamic state of the infection that allows us identifying transient and stationary states.

A culture is considered to be in a stationary state when two conditions are fulfilled:

- the mean age of RBCs in cultures varies according to the constraints imposed by the storage protocol 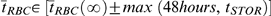, and
- the instantaneous diversity of IRBCs is maintained within the region: 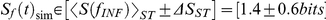.

**Figure 7:**
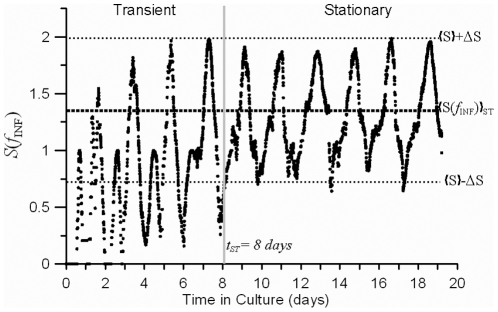
Simulated evolution of the instantaneous diversity index of infection forms *S_f_(t)*. *S_f_(t)* is compared to the prevalent diversity observed in experimental cultures *<S(f_INF_)>_ST_*. During the stationary state (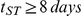), *S_f_(t)* is circumscribed within the standard deviation ΔS found when examining several experimental cultures operating at standard conditions.

According to the model outcome, we can distinguish two regions in the evolution of 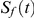 (Figure 7). After a certain amount of time 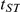, 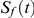 oscillates around an average value that does not significantly differ from 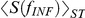 with a limited amplitude circumscribed within the region 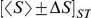.

### Factors limiting the diversity of the cultures

Over 200 simulations have been carried out varying three external factors that may affect the population structure of RBCs and IRBCs in culture: the storage period of RBCs prior to cultivation 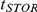, the degree of synchronism of the inoculum 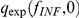 and the sub-cultivation protocols.

The analysis of the obtained results show that the population structure of RBCs in the stationary state is governed by 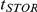 and by the sub-cultivation protocol, while the population structure of IRBCs in the stationary stage is not governed by the tested external factors. Nevertheless, these external constraints do determine the mean parasitaemia 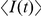, the average growth ratio of the infection 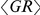 and the duration of the transient stage 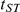.

The stationary state is achieved after a week in culture, on average, but simulations show great variability in their outcomes in the range of explored values. The time lag 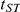 increases with the degree of synchronism of the inoculum (i.e. the sharpness of *q*(*t_INF_*)), with the duration of the sub-cultivation periods and with 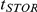, being the degree of synchronism of the inoculum is the most relevant factor. These results are outlined in tables shown in the Supporting Information file Chart S1.

It must be stressed that the prevalent regime achieved by *Pf* infections in static *in vitro* culture systems is not a stationary state but an oscillating one. Its characteristic features (for instance, number of cells, concentration of substrate, age of RBCs, post-invasion time of IRBCs) vary periodically. The characteristic periods and amplitudes of these oscillations are imposed both by the intrinsic infection dynamics and by the external constraints imposed on the system.

## Discussion

The design of *in vitro* culture systems, their analysis and the prediction of their performance consider their thermodynamic constraints even if these are not stated explicitly. These limitations are usually expressed as heuristic laws governing the system on the whole. In contrast, the joint application of a bottom-up description and an explicit use of thermodynamic concepts permits a clearer picture of the culture system and a better detection of misconceptions. Some practical implications regarding the static *in vitro* cultivation of *Plasmodium falciparum* extracted from the results here presented are discussed next.

First and foremost, cultures are always subject to thermodynamic constraints. It should be kept in mind that in static cultures these restrictions must be fulfilled at a local level as well as on the system as a whole. For instance, it has been claimed that unrestricted parasitaemia yields can be obtained by increasing the fraction of medium per RBC [25] (MR4, 2008), but this could not be experimentally reproduced by our group. According to our model, these results presented in the MR4 report are in contradiction with the diffusion limitations in the RBC layer [27] but such limitations can be avoided with alternative cultivation methods, such as hollow-fiber bioreactors [41] in static cultivation, or automated suspended sub-cultivation protocols such as the ones here proposed.

 Second, cultures maintained under fixed external constraints tend towards steady oscillating asynchronous infections after a transient lag. The diversity of infection forms in the stationary state is smaller than the one assuming maximum evenness of the population and it is independent of both the degree of synchronism of the inoculum and of the sub-cultivation protocol.

These two facts indicate that the infection process imposes intrinsic constraints that shape the population structure of IRBCs. In other words, in monoclonal static cultivation of *Pf* with a fixed strain of the parasite, 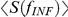 depends on the dynamics of the infection process alone. According to the model, the intrinsic factors that affect 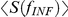 reflect the characteristic properties of the strain, such as the individual variability in the duration of the infection cycle, the endurance of the extracellular forms and the capacity of invasion of healthy RBCs. In contrast, the prevalent distribution of IRBCs 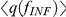 is not significantly affected by the degree of synchronism of the inoculum, nor by the sub-cultivation protocol. One can think of external factors that influence 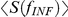, for instance, a treatment that specifically targets mature forms of the parasite. The experiments and simulations here presented did not explore such influences.

The results here presented also suggest that a stationary state with no oscillations is unlikely to be achieved in *Pf* cultivation *in vitro*. In theory, a strictly stationary state could be achieved if periodic variations were removed from external constraints. For instance, the 48-hour infection cycle periodic oscillations in the shape of 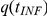 would disappear if invasion of healthy RBCs occurred at a constant rate, which would be the case if 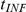 was evenly distributed among IRBCs. Similarly, oscillations in the total number of cells, in the age of RBCs and in the concentration of substrate would be abated if the culture was continuously renewed to maintain optimal conditions. In practice, the description above corresponds to the basic operation of a chemostat.

Several attempts have been made to design a steady bioreactor for culturing malaria since the first continuous cultivation, but to date none has managed to avoid discrete sub-cultivation under human supervision [42], [32], [43]. Beyond technical difficulties, INDISIM-RBC simulations suggest that infection dynamics of the culture make the achievement of a stationary state in the dynamics of 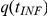 unlikely (results not shown). The individual variability of the infection cycle makes it very difficult to control the degree of synchronism of IRBCs and consequently hinders tuning such a device while the high multiplication rate of the parasite rapidly induces amplification of perturbations in the parasitaemia.

According to INDISIM-RBC, maintenance of stable malaria cultures in a continuous stirred-tank reactor with continuous renewal would require the continual readjustment of the input and output flows of RBCs in order to control fluctuations in the parasitaemia (results not shown). This hinders the development of chemostats with continuous and fixed input and output flows. Alternatively, we propose an automated method with discrete sub-cultivations, and we propose a criterion for comparing different samples of such system: to consider differences among cultures to be significant when the instantaneous diversities of infection forms differ in more than the standard deviation of the culture under standard conditions.

As shown in Figure 3, discrete medium renewal and sub-cultivation allows maintaining a bounded oscillating parasitaemia.

And third, the distinction between different dynamic states of cultures (transient and stationary) is crucial to assess the efficiency of drug treatments. In a drug trial, it must be granted that the tested and control systems are alike, that they only differ in the application of the drug and that they would behave similarly otherwise. This can be achieved through two strategies: granting that both systems are in a stationary state, or granting that both systems are in the same instant of a transient state. The results here presented may suppose an aid to the first strategy, as we propose a quantitative criterion to decide at least when systems are not in a stationary state: to compute 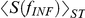 and 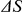 for similar cultures previously carried out and to check if the diversity of the culture under study 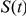 fulfils: 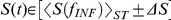. The second strategy is followed through synchronization of cultures with external manipulation [41].

The main motivation for using thermodynamic jargon is to take profit of its detailed definitions of fundamental concepts such as “system”, “scale” or “state”. The results here presented allow for a generalization to other microbial systems, models and sets of measurements. Some general considerations that arise from the use of IbM under a thermodynamic perspective are presented below.

First, given that a model is always a simplified representation of reality, the diversity index may be used as an indicator of how incomplete is the picture it offers, thus helping researchers to evaluate soundess of the model structure. To illustrate this, the scheme of operation followed in this paper is presented below using a non-specific terminology.

Basically, we can assume that any population is characterized by a set of individual features (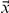) distributed among the population, that this distribution (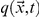) varies with time and that it can be computed to obtain the instantaneous diversity of the community at any time 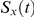.

On the one hand, 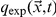 can be monitorized in experiments. If regular patterns are found in the temporal evolution of the system, the prevalent diversity associated to those patterns 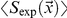 can be defined. On the other hand, 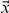 can be used to build a model that defines a set of rules describing the evolution of 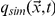. The diversity of 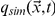 may be maximized assuming the constraints of the model to obtain its maximum expected value 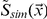.

Then, 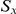 can be used to evaluate the structure of the model, or at least, to assess its capacity to account for the observed diversity. If 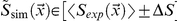, we can assert that the model is sound. Otherwise, the structure of the model does not fit observations and it should be revised.

Finally, this criterion is not a unique indicator of the goodness of a model, but a test that should be performed on models together with other examinations. The analysis here presented can be applied to various sets of individual features and to multiple patterns, and it can be considered in a framework of pattern-oriented modelling, a strategy adopted to build the simplest structurally realistic models that capture the relevant behaviour of complex systems in ecological modelling [12], [44].

And second, 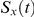 can be used for comparing different systems or different samples of the same system, in order to determine their dynamical state. In particular, 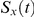 sampled at any time can be compared to the diversity of the stationary state 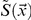 (if known) or to the observed prevalent diversity of the system 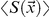. The threshold of similarity between the instantaneous and reference diversities 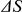 may be extracted from the experimental variability of the system 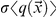. This comparison provides a criterion to assess whether a microbial community is undergoing a transient stage or has reached a stationary state.

As a closing remark, the analysis here presented may be appropriate to address specific problems regarding the organization of other aspects of *Pf* cultivation, such as gametocytogenesis, in particular, and of microbial populations, in general. It may be suitable to deal with those systems where cellular diversity plays a central role, *e.g*.: characterization of the collective stages of microbial growth [45], as well as to tackle those systems in which the spatial structure shows permanent or attractant patterns, *e.g.*: flocking transitions [46], and to analyse the evolution towards stable regimes of microbial populations, in general, *e.g.*: ecological succession or collective auto-organization [47].

## Supporting Information

Text S1 **Experimental trials of the** ***in vitro*** **cultivation of** ***Plasmodium falciparum*** **infected human Red Blood Cells.** Outlined description of some specific experiments carried out by the Experimental Microbiology Group from GlaxoSmithKline during the period 2005-2009.
(DOC)

Text S2 **Description of the computational model and simulator.** INDISIM-RBC is described following the guidelines of a standard protocol to describe Agent-based Models.
(DOC)

Chart S1 **Outcome of INDISIM-RBC simulations.** A set of tables list the input parameters representing different initial conditions of the simulated cultures and their corresponding outcome.
(XLS)
