# Supplementary material for: Thermodynamic Concepts in the Study of Microbial Populations: Age Structure in Plasmodium falciparum Infected Red Blood Cells
Source: PLoS One. 2011 Oct 31;6(10):e26690. doi: 10.1371/journal.pone.0026690 (PMC3204994; doi:10.1371/journal.pone.0026690)
Supplement: Text S1 — Experimental trials of the in vitro cultivation of Plasmodium falciparum infected human Red Blood Cells. Outlined description of some specific experiments carried out by the Experimental Microbiology Group from GlaxoSmithKline during the period 2005-2009. (DOC) [file pone.0026690.s001.doc]

APPENDIX I: SPECIFIC MODELS AND EXPERIMENTAL TRIALS OF THE *IN VITRO* CULTIVATION OF *Plasmodium falciparum* INFECTED RED BLOOD CELLS

MODEL USED TO ASSESS THE EFFECT OF STORAGE OF RBCS

Several studies indicate that *P. falciparum* has a preference for infecting young RBCs. Firstly, age fractionation of RBC populations prior to cultivations shows that the growth ratios of the infection are significantly greater for those cultures performed with samples containing more retyculocites (young RBCs aged < 2-6 days) [Pasvol et al 1980]. Secondly, studies of multiple infections confirm that the susceptibility to invasion of retyculocytes is 4-fold the susceptibility of older RBCs [Simpson et al 1999].

Human RBCs for malaria cultures are usually obtained from blood that is considered not to be usable for blood transfusions. This blood can be kept frozen (at temperatures that may reach -80ºC) for several years without lost of harvest viability. Cultures with blood frozen for 19 months and 24 months do not show significant differences when compared with control cultures from fresh blood [Pavanand et al 1974].

In addition, whatever the source and manner of storage of RBCs, they must be prepared prior to the cultivation. They are washed and mixed with culture medium and usually subsequently stored at temperatures that range from 1ºC to 4ºC, prior to the cultivation. The time spent in these conditions affects the culture's performance. The blood storage regimes have influence on the RBC suitability when storage exceeds 3 or 4 weeks [Capps and Jensen 1983]. Cells can be used for up to 5 weeks of storage at 4ºC and are typically maintained for 4 days to a week once washed [Trager 1994].

To sum up, storage spans greater than the age of retyculocytes have no significant effect on RBC susceptibility to infection, but the performance of the infection still depends on this storage. Besides, significant differences in the parasite proliferation are observed between cultures of young and old RBCs.

The hypotheses assumed in the model are:

1. the parasite has a preference for young cells, and also detects their freshness, and
2. storage ages RBCs, reducing their freshness but without changing them. Presumably, extremely low temperatures prevent RBC maturation.

These assumptions are introduced in the model of the temporal evolution of $p_{INF}$ in two ways:

1. as the abrupt decay in $p_{INF}$ after $t_{RBC}> 40 days$, and
2. as a linear decay of $p_{INF}$ from $p_{INFMAX}=0.8$ at day 1 until reaching a minimum value $p_{INFMIN}=0$ at day 120. This linear decay is truncated by 1.

The trial of virtual experiments used to test this model consists in launching simulations of the *candle-jar* [Trager and Jensen 1976] cultivation of the parasite where the subcultures are fed with RBCs that proceed from sources stored for different time spans. Two series of static cultures are simulated, with sub-cultivations that set the parasitaemia at the new culture to 0.5%, each 48 h and each 96 h, respectively. Initial parasitaemia is set to $I_{0}=0.5$.

Each trial explores culturing protocols where the sub-cultivation are carried out using increasingly old RBCs (Storage spans are set to: 0 days, 4 days, 8 days, 12 days, and up to 3 months). The average parasitaemia of long-term cultures maintained for up to 3 months is compared for the different renewal protocols.

MODEL USED TO DESIGN AN AUTOMATED SUB-CULTURING PROTOCOL

Apparently, suspended cultures show increased infection growth ratios [Butcher, 1981 and 1982] and support higher parasite yields [Zolg et al, 1982] without an excessive increase in the medium expenses [Fairlamb et al 1985].

Taking into account the local limitations on parasite spreading, the performance of static cultures on the whole is limited by the spreading of the parasite through the hematocrit layer and by the diffusion of substrate from the medium that is soaking the settled RBCs [Ferrer et al, 2008]. An empirical model consistent with these constraints can be adjusted to the behaviour of the infection on the whole.

The model assumes unrestricted growth of the infection in the short term (for periods equal or smaller than 48 hours to exclude successive infection cycles), which leads to an exponential increase of the number of IRBCs: $N_{IRBCs}(t+1)=N_{IRBCs}(t+1)\cdot e^{\alpha t}$ .

The value of the growth rate $\alpha$ reflecs the average availability of healthy RBCs at merozoite release after the infection cycle. It is obtained by averaging the daily growth ratio of the parasite over the whole culture system during a 10-day cultivation $\left\langle GR\right\rangle_{24}$ and by fiiting Equation 1, measuring time in hours.

The values of the parameters in the model that control RBC susceptibility to invasion, spreading rate of merozoites and cell density are adjusted to reflect the culturing conditions and to fit the experimental values of $\left\langle GR\right\rangle_{24}$.

For a static culture, we find $\left\langle GR\right\rangle_{24}^{static}=1.8\pm0.6$ and we adjust .

Meanwhile, the expected behavior of the suspension systems is a compromise between two opposite trends:

On the one hand, the continual shift of positions of the RBCs favors the propagation of the disease because the number of potentially healthy RBC targets to be invaded by an egressed merozoite is no longer restricted to the immediate neighborhood of the lysing IRBC, but . In addition, the mix of the hematocrit layer homogenizes the concentration of solute substances, thereby eliminating the effect of local substrate limitations. As a result, these effects tend to increase the growth ratio of the infection.

On the other hand, the reduction of cell density and the continual movement of RBCs hinder the invasion of fresh RBCs in suspended cultures. This can be understood as the decrease in the probability of encounter between a merozoite and RBC and to the presumably increased difficulty of attachment to a moving cell. In addition, the death probability is likely to be increased when the RBCs are submitted to the damaging methods usually employed to maintain cells in suspension (*i.e.* airlift columns or stirring, shaking or wiggling of the culture systems). For a culture with a RBC concentration of 5%, we find: $\left\langle GR\right\rangle_{24}^{stirred}=3.7\pm1.7$ and we adjust $\alpha_{A}=0.05 h^{-1}$.

The above arguments suggest that the agitation of static culture systems at discrete time spans would increase the performance of static *in vitro* cultivation of *P. falciparum*. Indeed, discrete gentle agitation of the hematocrit combines the increase in the number of potential healthy RBC targets with the high cell densities within the hematocrit layer, atking advantage of both strategies, leading to the value: $\alpha_{C}=0.09 h^{-1}$.

Static cultures are represented by INDISIM-RBC as described in the Appendix II. Cultures in suspension are modeled as a custom static model with two modifications. First, the system is continuously mixed: at each time step, the position of the RBCs is shifted at random, merozoites are scattered all through the spatial cells and substrate is continuously homogenized. And second, the RBC density in the model is set in correspondence to the volumetric hematocrit concentration (we have tested concentrations ranging from 5% to 10% in volume) rather than to the cell density observed in the hematocrit layer (where around 80% of the volume is occupied by RBCs). This marked reduction in cell density is introduced in the model as a decrease in the total number of modeled RBCs.

Discretely agitated culture systems are represented by INDISIM-RBC as static cultures where the positions of RBCs are periodically shifted at each agitation. INDISIM-RBC shows no differences between the behavior of static cultures and discretely agitated cultures with different agitation periods. The results obtained in real and simulated cultures suggest that either the local limitations (regarding the spreading of the parasite and/or the concentration of substances in solution) are not overcome with discrete agitation, or that there are no such local limitations.

EXPERIMENTAL CULTURING PROTOCOLS

The simulations here presented have been compared to the experimental work carried out by the Experimental Microbiology Group from GlaxoSmithKline (EMG-GSK). *P. falciparum* infected RBCs *in vitro* cultures were raised under the culturing conditions described by the protocols published in 2005, and revised in 2008, by the Malaria Research and Reference Reagent Resource Centre (MR4). A schematic description of the culturing protocols is outlined in Table 1.


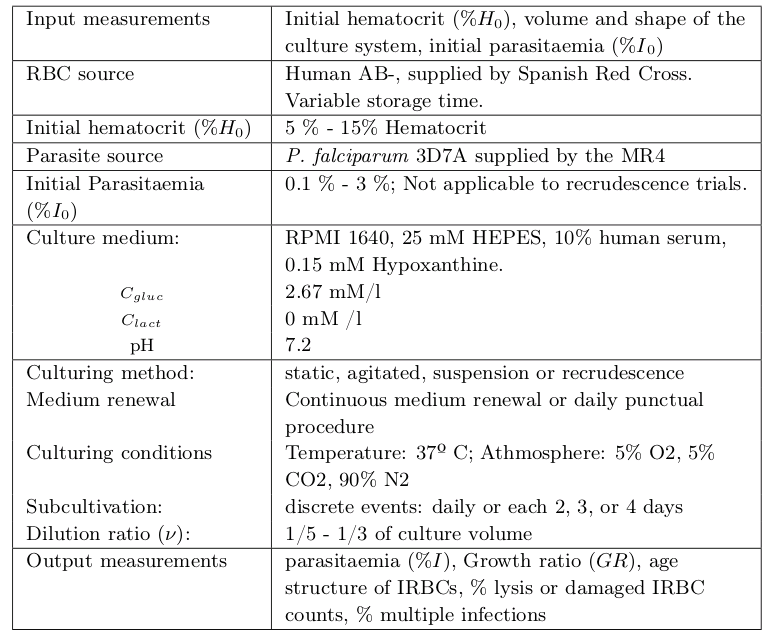


Table 1: General conditions of the cultures performed by the EMG-GSK. Detailed specification sheets for RPMI1640 culturing medium and for human serum may be found at commercial suppliers.

Some specific trials mentioned in the manuscript are explained with grater detail in next section. Experimental trials that have been used to calibrate the model or to test hypothesis beyond the scope of this paper, but which have been used here 1) to evaluate the variability of different culture with respect to the initial conditions and 2) to evaluate the variability of the prevalent diversity in static cultivation are outlined in a miscellaneous category for informative purposes,.

SPECIFIC EXPERIMENTAL TRIALS

1. Parasite strain 3D7 in static and suspended cultures

> Outline: In order to study the variability of its performance, continuous cultivation of *P. falciparum* 3D7 strain in static and suspended cultures is set with different strains of the parasite.

> Experimental methods: The experiment comprises 18 (10-days) trial runs comparing static and suspended cultures with the same culturing protocol and initial conditions (detailed in Table 1, with 3-day sub-cultivation). Differences in the culture yields are assessed by measuring the prevalent parasitaemia and the average growth ratio at each sub-cultivation.

> Conclusions: The intrinsic variance of the performance of each trail is of the order of 5%. The intrinsic variability of each parasite strain is of the order of 50% when the static and suspended methods are compared. No significant correlations are found between the performances of each culture method. Some paraite strains seem to grow better in suspension while other show better yield in static cultivation.

1. Renovation and sub-cultivation regimes in static cultivation

> Outline: In order to study the viability of continuous cultivation of the parasite, *P. falciparum* is cultured under static conditions with two actuation patterns: simultaneous medium renewal and subcultivation of the hematocrit every 48 hours and daily medium renewal with dilution of cultures every 72 hours.

> Experimental methods: The experiment comprises four (10-days) trial runs comparing two different culturing protocols with fixed medium renewal and subcultivation periods. Medium renewal replaces 100% of the culture medium and the dilution fraction at subcultivation is variable and set to obtain a resulting parasitaemia of *I* = 0.5%. Specifications are listed in Table 2.


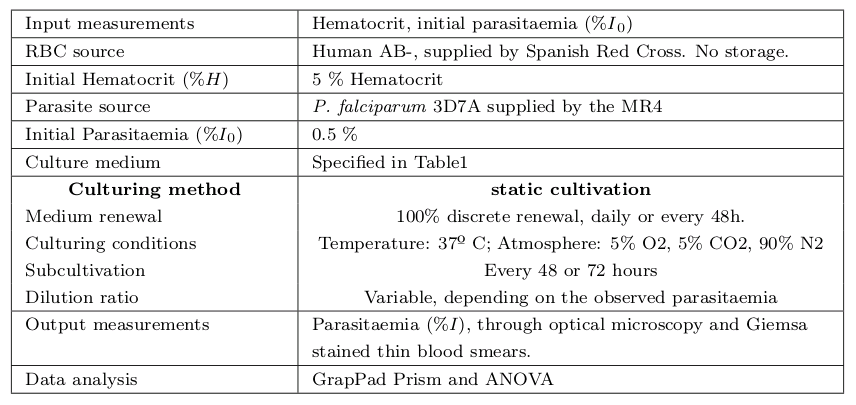


Table 2: Specifications of the experimental procedure for the comparison of different short-term cultivation regimes.

> Conclusions: No significant differences are found between the cultures with a 48 hour-period for both medium renewal and sub-cultivation, and those cultures with daily medium renewal and sub-cultivations every 72 hours or every 96 hours.

1. Sub-cultivation regimes in automatic cultivation

> Outline: In order to determine the appropriate dilution of subcultures for the continuous automatic cultivation of the parasite, P. falciparum is cultured with two agitation patterns: under static undisturbed cultivation regime and under suspension culturing conditions, through the continuous agitation of the hematocrit with a magnetic stirrer.

> Experimental methods: The experiment comprises four (10-days) trial runs comparing two different culturing protocols with fixed medium renewal and automated sub-cultivation periods. Medium renewal replaces 100% of the culture medium and the dilution fraction at sub-cultivation is variable and set to obtain a resulting parasitaemia of *I =* 0.5%.

Samples of V = 5 ml of culture are maintained in glass bottles of 20 cm² base surface. Cultures can be maintained in suspension with the use of a magnetic stirrer operating at low velocity. Parasitaemia is measured daily through the extraction of samples with minimal perturbation of the culture. Daily parasitaemia measurements of the samples extracted from the two replica of each trial are compared with ANOVA. The specific experimental protocols are summarized in Table 3.

> Results: The values were used to define the renovation factors at sub-cultivation $\nu_{A}$ and $\nu_{B}$ were obtained from the average growth ratio observed between successive sub-cultivations. The average 48h multiplication ratio for static cultures obtained was $\left\langle GR\right\rangle_{48h}^{static}=3.06\pm1.06$ while the multiplication ratio for stirred cultures was $\left\langle GR\right\rangle_{48h}^{stirred}=11.16\pm2.1$. At the beginning of 4^th^ cycle, static cultures do not growth, probably because the low surface of bottles compared with the surface of flasks, and because the shape of these bottles that have got a concavity surface. In case of stirred cultures, the possible reason of the decrease of growth, could be assigned to high levels of parasitemia obtained on day 11^th^ so many stressed forms of parasite were detected.


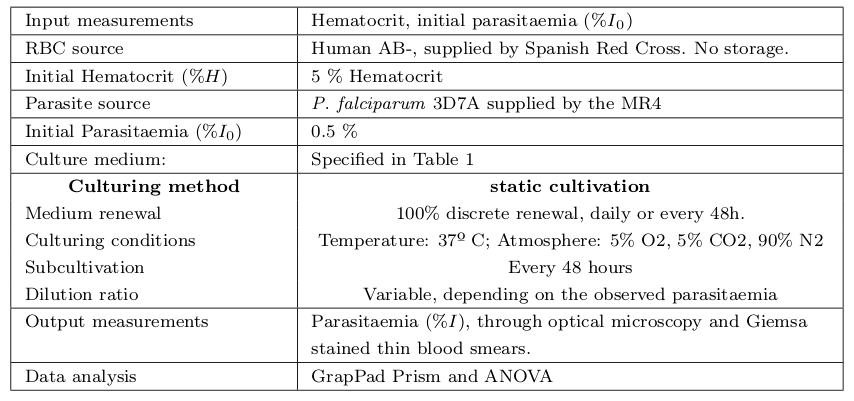
Table 3: Specifications of the experimental procedure for the comparison of different long-term cultivation regimes.

> Conclusions:

1. Static culture systems can be continuously maintained during 5 cycles when the fraction of subcultured cells to the fresh healthy RBCs at sub-cultivation is equal to $\nu=\frac{1}{3}$.
2. Stirred culture systems can be continuously maintained during 5 cycles when the fraction of subcultured cells to the fresh healthy RBCs at sub-cultivation is equal to $\nu=\frac{1}{11}$.

* Agitated cultures with discrete activation of the stirrer were not carried out.

1. Miscellaneous

> In order to calibrate INDISIM-RBC, we used data from literature (Pavanand et al 1974, Trager and Jensen 1976, Butcher 1981 and 1982, among others). Their published data has been used to calibrate the variability of the model outcome.

> In order to evaluate the yield of different commercial culture mediums, 18 (10-days) trial runs have been carried out. Data from these experiments have been used here uncorrelatedly to evaluate the variability related to the initial distribution of IRBCs and to the prevalent diversity in static cultures.

> In order to evaluate the effect of different agitation regimes in suspended cultures 5 short-term (3-days) closed experimental trials have been carried out. Data from these experiments have been used here uncorrelatedly to compute the variability related to the initial distribution of IRBCs and to the prevalent diversity in suspended cultures.

> In order to evaluate the effect of global substrate availability, three short-term (3-days) closed experimental trials have been carried out with different concentration of glucose. Data from these experiments have been used here uncorrelatedly to evaluate the variability related to the initial distribution of IRBCs and to the prevalent diversity in static cultures.

To sum up, we have used data of 29 10-day experimental trials to determine the prevalent diversity of static cultures: 4 MR4 standard cultures, 9 cultures that had been used to evaluate different commercial sources of culturing medium, 9 cultures that were used to study the variability of different inocula of the 3D7 strain, 3 (short term experiments) that had been used to explore the effect of glucose concentration, 2 that explored different static manual sub-cultivation protocols and 2 that had been used to study automated sub-cultivation protocol. Observations from these systems did not show significant differences when compared to 30-day cultivations. Each of these experimental trials was reproduced with the corresponding simulation. We also performed 10 simulations for each scenario of initial conditions and variations of the standard protocol for static cultivation, a total of to 200 runs (see Supporting Information files S2 and S3).

REFERENCES:

Butcher G.A (1981) A comparison of static thin layer and suspension cultures for the maintenance in vitro of Plasmodium falciparum. Ann. Trop. Med. Parasitol. 75 (1), 7-17.

Butcher GA (1982) The behavior of different strain of Plasmodium falciparum in suspension and static cultures. Trans. Roy. Soc. Trop. Med. Hyg. 76 (3), 407-409.

Capps TC, Jensen JB (1983) Storage requirements for erythrocytes used to culture Plasmodium falciparum. J. Prasitol. 69 (1),158-162.

Ferrer J, Rosal MD, Vidal JM, Prats C, Valls J, *et al.* (2008) Effect of the hematocrit layer geometry on Plasmodium falciparum static thin-layer in vitro cultures. Malaria Journal 7: 203.

Pasvol G., Weatherall DJ, Wilson RJM (1980) The increased susceptibility of red blood cells to invasion by the parasite Plasmodium falciparum. Brit. J. Haematol. 45, 285-295.

Pavanand K, Permpani B, Chuanak N, Sookto P, (1974) Preservation of Plasmodium-falciparum-infected erythrocytes for in vitro cultures. J. Parasitol. 60 (3), 537-539.

Simpson JA, Silamut K, Chotivanich K, Pukrittayakamee S,White NJ (1999). Red cell selectivity in malaria: a study of multiple-infected erythrocytes. Trans. Ry. Soc Trop. Med. Hyg. 93 (2), 165-168.

Trager W (1994) Cultivation of Malaria parasites. Meth. cell. biol. 45, 7-26.

Trager W, Jensen JB (1976) Human malaria parasites in continuous culture. Science 193, 673-675.

Zolg JW, MacLeod AJ, Dickson IH, Scaife JG. (1982) Plasmodium falciparum: modifications of the in vitro culture conditions improving parasitic yields. J. Parasitol. 68 (6), 1072-1080.
