# Supplementary material for: Thermodynamic Concepts in the Study of Microbial Populations: Age Structure in Plasmodium falciparum Infected Red Blood Cells
Source: PLoS One. 2011 Oct 31;6(10):e26690. doi: 10.1371/journal.pone.0026690 (PMC3204994; doi:10.1371/journal.pone.0026690)
Supplement: Text S2 — Description of the computational model and simulator. INDISIM-RBC is described following the guidelines of a standard protocol to describe Agent-based Models. (DOC) [file pone.0026690.s002.doc]

APPENDIX II: INDDISIM-RBC DESCRIPTION ACCORDING TO THE ODD PROTOCOL

The ODD protocol is the standardized protocol used to present Individual-based Models in ecology in order to facilitate their communication and sharing (Grimm et al, 2006).

PURPOSE

The model studies the the relation between population structure and infection dynamics in the cultivation of *Plasmodium falciparum* *in vitro*.

CHARACTERSISTIC SCALES

The model reproduces a small fraction of an experimental culture system under standard static conditions: a vertical cut of the hematocrit layer - settled Red Blood Cells (RBCs) soaked in culture medium- of (0.1mm x 0.1 mm) base and 0.5 mm height. The space is modeled as a regular grid formed by cubic spatial cells of side d=5*μ*m. Processes are at discrete regular events. The time step is set to correctly describe the propagation of the extracellular parasite through the hematocrit layer: *ts* = 6 minutes. The simulation unit to represent substrate particles stands for 1*su* = 10^5^ molecules of nutrient.

STATE VARIABLES:

The model comprises two low level entities: the RBC and its local environment, the spatial cell. The extracellular parasite (merozoite) can also be regarded as a low level entity, although it has not individual characteristics apart from its location in the spatial grid and age.

> The RBC is the red blood cell susceptible to infection

Global characteristics (common for all RBCs):

- volume,
- density,
- mean uptake rate.

Individual characteristics (set when the RBC is defined in the model):

- maximum age ($t_{RBCMAX}$[ts]): the maximum number of time steps that can be maintained in storing conditions.
- duration of the infection cycle ($t_{INFMAX}$ [ts]): duration of the intra-erythrocytic cycle of the parasite. Duration of each of the infection stages during the infection cycle.
- accidental death rate per time step curve ($p_{death}$).

Individual variables (vary through the simulation):

- location in the spatial grid,
- age ($t_{RBC}$[ts]), includes the storage prior to cultivatio,
- infection stage ($f_{INF}$-0:healthy, 1: ring, 2: trophozoite, 3: early schizont, 4: late schizont -),
- post-invasion time ($t_{INF}$[ts] – 0: healthy, infection cycle: from 1 to 55 hours-),
- metabolic stress due to lack of glucose or excess of lactate,$\text{IM}$.

> The merozoite refers to the extracellular form of the parasite:

- location in the spatial grid,
- time steps in the extracellular medium ($t_{mero}$[ts])

> The spatial cell:

- fraction of volume not occupied by RBCs and merozoites ($0<f<1$,
- number of RBCs of each infection stage,
- number of extracellular merozoites of each age (CMEROCLXYZ).
- amount of glucose ( $C_{gluc}$[*su*])
- amount of lactate ($C_{lact}$[su])

PROCESS OVERVIEW

At each time step, many processes affect the low level entities of the model.

> Processes affecting the RBC:

- *Motion.* RBCs may eventually fall down to lower positions in the hematocrit layer.
- *Uptake.* each RBC uptakes an amount of nutrient that varies with the post-invasion times for IRBCs.
- *Infection.* i) Healthy RBCs may be infected by a merozoite placed in its same spatial cell with a preset probability. This probability decreases with RBC age. There is a probability of being infected by more than one merozoite.
  ii) Infected RBCs follow the infection cycle and may not be infected again. At the end of the infection cycle, the IRBC dies, breaks out and releases from 8 to 16 merozoites. The released forms are placed at random in the same spatial cell and in the nearest-neighbouring cells.
- *Death.* RBC may die due to many reasons: accidental death, exceeding the maximum age or exceeding the maximum stress index. Mature IRBCs have an increased accidental death rate.

> Process affecting the merozoites:

- *Spreading.* At each time step, the merozoite randomly moves to nearest-neigbouring cells.
- *Invasion.* A merozoite may invade any healthy RBC in the same spatial cell.
- *Inactivation.* Occurs after 5 *ts* the merozoite is no longer invasive.

> Processes affecting the spatial cells:

- *Diffusion.* Substrate propagates from cell to cell following the Fick’s law to compensate concentration gradients. The total surface between cells is not taken into account.
- *Motion / death of RBCs*. RBCs may enter or abandon any spatial cell.
- *Merozoites.* Merozoites may enter or abandon any spatial cell.

> Process affecting the culture system on the whole:

- *Sub-cultivation*. The population of RBCs is renewed periodically, each 2, 3 or 4 days.
- *Medium* renewal. Occurs daily or two days after a sub-cultivation. A fraction of the merozoites are removed. Concentration of glucose and lactate is set to its initial values
- *Agitation*. Position of every RBC and merozoite is changed at random.

SCHEDULING:

The general scheduling of the program is: setting of the initial configuration of the model, simulation of the model evolution during a fixed number of time steps or until some conditions are achieved, and end of simulation.

At each time step, first, all the actions of the individuals take place. Individuals act sequentially but the order in which they act changes at each time step. Individual actions affect solely the spatial cell in which they are. The changes in the spatial cell due to individual processes are updated as the individual action occurs. The order of individual actions is: *motion, uptake, infection death*.

Then, the local actions of the spatial cells are modeled in this order: spreading of merozoites and diffusion of substrate. The state of each spatial cell and its first neighbors determines each action. The state of all the spatial cells is updated at the same time, after each one has acted Actions on the whole system entail a partial or total reset of the variables of each spatial cell and individual. First, agitation is checked, then sub-cultivation and finally, medium renewal. At the end of each time step, relevant information is stored in output files and visualized on the fly. At the end of the simulation, the output s are analyzed.

DESIGN CONCEPTS:

*Emergence.* Population structure (distribution of post-invasion times among the IRBC population) emerges from the individual defined infection cycles, from the local interactions among RBCs and merozoites and from the stochasticity. Different infection proliferation rates within the culture, related to the population structure (synchronic and asynchronic cultures) also emerge from the individually defined rules. The limitations on the proliferation of the infection for models of small culture systems (layers with depths below 0.2 mm) emerge from the spreading of the parasite. Limitations for thick hematocrit layers are expected to be obtained from substrate diffusion limitations, however they are currently introduced as externally imposed system-level rules. Other patterns observed in the infection dynamics are imposed at a system level, according to the protocols described in the experimental manipulation of the culture system.

*Sensing, prediction, fitness seeking and adaptation.* Individuals don’t sense their environmental conditions and have no choice to act in response to what they are sensing. They do not search for fitness and no adaptation rules are defined.

*Stochasticity.* Randomness is introduced in two ways. First, we consider heterogeneity in the population, a random distribution of individual characteristics among the population. Second, uncertainty and variability are introduced at many phenomena occurring at a cellular level as Gaussian noise on the expected values or as probabilistic events. Random numbers are also used to create the tables that determine the order of action when many entities are going to act sequentially.

*Interactions.* The main interaction between individuals (infection) is carried out via the merozoites. RBCs also interact with each other by competing for nutrient, and accumulating harmful substances. They also interact with each other through the occupancy of empty spaces in the hematocrit layer.

INITIALIZATION:

Cultures are set with spatially homogeneous distributions of RBCs, infected RBCs and nutrients. Each spatial cell can be occupied by one RBC at most, therefore the spatial grid has a number of cells equal or greater than the initial population of RBCs. No merozoites are initially distributed. Dimensions of the spatial grid, initial parasitaemia, total number of RBCs, and age and population structure of the inoculum are specifically set to reproduce experimental behaviors. Initial data are taken from literature.

Common characteristics:

- volume = 83 μm^3^
- density = 1.1 density of the medium
- glucose uptake rate when healthy = 2x10^6^ molecules of glucose/minute = 20 *su/ts*. Uptake rate varies with the post-invasion time for IRBCs, reaching 100-fold values.

Individual characteristics (set when the RBC is introduced into the model):

- $t_{RBCMAX}$is set following Gaussian distribution with 10% variance around the observed mean duration of RBCs in storage conditions (30-40 days),
- $t_{INFMAX}$Is set following a Gaussian distribution with 10% variance around 48 hours. Duration of each of the infection stages during the infection cycle are set accordingly (ring: 18 hours, trophozoite : 17 hours, early schizont: 7 hours, late schizont: 6 hours,
- $p_{death}$ takes values that depend on the infection state of the IRBC. (healthy, rings: 0, trophozoite. 0.001, early schizont and late schizont: 0.003).

Spatial cell:

- fraction of the hematocrit layer occupied by RBCs 0.65,
- diffusion coefficients for substrate D_gluc_=3.16·10^-6^ cm^2^/s and D_gluc_=2.6·10^-6^ cm^2^/s, respectively,
- substrate initial concentration. C_gluc_=2.677g/l. C_lac_= 0 g/l.

The boundary conditions of the space are:

- open boundary conditions for the upper spatial cell layer. We define an extra layer with a constant concentration of glucose C_gluc_ =2.677 g/l and lactate C_lac_= 0 g/l,
- closed boundary conditions for the lower layer. No diffusion, no motion is allowed,
- periodic boundary conditions for the side spatial cells.

SUBMODELS:

*Shuffling:* Many of the actions that are performed by the individual entities (RBC and spatial cells) take place sequentially. In order to reduce the artificial bias on the model outcome caused by the process of sequentation, we impose random orders of action for the entities. This means that the other in which the entities act changes at each sequence of actions, therefore no entity is always favored with he first turn of action. The way to perform this randomization of the sequences is to initially create and store several disordering tables that will reshuffle the given lists of RBCs, merozoites or spatial cells with minimum computation time. This allows not generating random numbers every time we need to disorder a list.

> Actions of the RBCs.

*RBC Motion.* RBCs have a density slightly higher that the density of the culture medium. Therefore they tend to settle down in a layer. The fall of RBCs through the hematocrit layer is modeled as follows: whenever there are no RBCs in the 9 cells immediately below the RBC, the RBC may fall to any of these cells. The probability of falling there at each time step can be controlled at will, but is normally fixed to 0.6, a value obtained from the falling terminal velocity in a viscous medium.

*RBC uptake.* At each time step, each RBCs attempts to uptake as much nutrient as it is required by the cell. The model supposes a constant uptake of a certain number of particles f glucose per time step (CON= 20-25 su/ts), with independence from the concentration of nutrient. At each time step the value CON for each RBC is chosen from a distribution centered on the mean observed uptake rate for RBCs. Whenever there is not enough nutrient to fulfill this uptake, the RBC undergoes a stress that entails more uptake requisites for the following steps. If the nutrient does not increase in the immediate next three ts, the RBC dies due to this stress. Infected RBCs have more energetic demands than healthy RBCs and it has been observed that they consume much more glucose. The uptake rate for IRBCs varies with the infection cycle and it has been observed that reaches values 100-fold the uptake of healthy RBCs. The model supposes that IRBCs have uptakes demands that vary with the post-invasion times (τ) in the following way: CON_IRBC_=CON_RBC_·*f(τ)*

1 ; τ<τ_R_  (Ring stage)

= 2·(τ−τ_R_−50) ; τ_R_<τ<τ_R_+50 (Tropozoite stage)

100 ; τ < τ_T_-50 (Early Schizont stage)

min(1, 100-2·(τ−τ_T_+50)) ; τ_T_-50<τ<τ_C_  (Late Schizont stage)

where τ_R_ , τ_T_ and τ_C_ are the durations of the ring stage, tropozoite stage and infection cycle respectively.

*RBC infection.*  At each time step, for each healthy RBC, the model counts how many merozoites are in the same spatial cell. For each merozoite in the same spatial cell, we evaluate its invasion of the RBC. The invasion is a probabilistic event that occurs with a probability . is a variable that decreases with the RBC age (it is gradually reduced a 0.01 % of its value per hour and rapidly drops after 40 days) and it takes values that depend on the parasite strain and on the blood source ). For a trial of experiments or simulations in the model this value has been maintained. Multiple invasion of RBCs are allowed, until reaching the maximum value of 3 parasites per IRBC.

*IRBC lysis.* At the end of the infection cycle, each RBC dies and “explodes”, and a variable number of merozoites are scattered into the surrounding medium. The total number of dumped merozoites depends on the duration of the last stage of the infection cycle (late schizont stage). This stage may last n=2,3,4 or 5 ts, and the duration is randomly distributed with a binomial probability distribution centered on 3.5. The total amount of merozoites dumped into the external medium is 2^n^.

For each of the merozoites, we randomly select the destination spatial cell from the same spatial cell and first neighbors.

*RBC death.* Each RBC may die due to accidental death, because it exceeded its maximum age, at the end of an infection cycle, or because it could not fulfill its metabolic demands. Whenever a RBC dies it is removed from the model. In its place, some remaining materials (representing the cell membrane and other structures) are left occupying a small volume (approx. 8% of the RBC volume). The remains of RBCs may fall through the hemaocrit layer in the same way as the RBCs do, but for them it is not a requisite that the cells below them contain no RBCs. These remains are removed from the model at each subcultivation or when the culture medium is renewed, when the cells of the olther culture are washed.

> Actions of the spatial cells.

*Merozoite propagation:* For each spatial cell, we count the number of merozoites in the same spatial cell. For each merozoite, we perform a random walk with the same probability of staying in the spatial cell as for moving to any neighbor cell. We also allow the possibility of moving a further cell downwards. In this sense, we allow the possibility of having a downwards-biased random walk.

*Substrate diffusion:*  For each spatial cell, at each time step, the model evaluates the difference in concentration with the neighbor cells and allows a partial balance of the concentration of substrate. Diffusion is carried out using an explicit Forward Euler Method. There is a maximum threshold for the amount of substrate that can be transferred per time step to a single neighbouring spatial cell to avoid numerical errors.

SIMULATIONS:

The model has been coded in Fortran 2003, and implemented with Compaq Visual Fortran V6.6 ®. The full source code, together with a sample simulation executable can be found at:

https://mosimbio.upc.edu/research-topics/biomedical-sciences/protozoa-malaria

REFERENCES:

Grimm V, Berger U, Bastiansen F, Eliassen S, Ginot V *et al.* (2006) A standard protocol for describing individual-based and agent-based models. *J. Ecol. Model.* 198 (1), 115-126.
